# Prevalence and adverse outcomes of pre-operative frailty in patients undergoing carotid artery revascularization: a meta-analysis

**DOI:** 10.3389/fcvm.2023.1297848

**Published:** 2023-11-28

**Authors:** Zeyu Liu, Ying Yao, Meiwan Zhang, Yan Ling, Xiaoyan Yao, Min Hu

**Affiliations:** ^1^ College of Nursing, Nan Chang University, Nan Chang, China; ^2^ Department of Neurology, The Second Affiliated Hospital of Nanchang University, Nan Chang, China

**Keywords:** Frailty, carotid artery stenosis, surgery, adverse outcome, meta-analysis

## Abstract

**Introduction:**

Frailty can lead to a decrease in the patient's resistance to interference such as injury and disease, and cause a series of complications. An increasing number of studies have found that pre-operative frailty exacerbates the occurrence of adverse events after carotid artery revascularization, but an integrated quantitative analysis is currently lacking. Therefore, we conducted a meta-analysis to evaluate the impact of pre-operative frailty on patients undergoing carotid artery revascularization.

**Method:**

According to the PRISMA guidelines, we systematically searched for relevant studies on Medline, Embase, Ovid, CINAHL, Web Of Science, and Cochrane Library from establishment until June 2023. Summarize the risk of adverse outcome events through OR and 95% CI.

**Results:**

A total of 16 cohort studies were included, including 1692338 patients. Among patients who underwent carotid artery revascularization surgery, the prevalence of pre-operative frailty was 36% (95% CI = 0.18–0.53, *P* < 0.001). Compared with non frail individuals, frail individuals have an increased risk of mortality (OR = 2.35, 95% CI = 1.40–3.92, *P* = 0.001, *I*^2^ = 94%), stroke (OR = 1.33, 95% CI = 1.10–1.61, *P* = 0.003, *I*^2^ = 71%), myocardial infarction (OR = 1.86, 95% CI = 1.51–2.30, *P* < 0.001, *I*^2^ = 61%), and non-home discharge (OR = 2.39, 95% CI = 1.85–3.09, *P* < 0.001, *I*^2^ = 63%).

**Conclusion:**

The results of this article show that patients undergoing carotid artery revascularization have a higher prevalence of pre-operative frailty, which can lead to an increased risk of postoperative death, stroke, myocardial infarction, and non-home discharge. Strengthening the assessment and management of frailty is of great significance for patient prognosis.

**Systematic Review Registration:**

https://www.crd.york.ac.uk/prospero/display_record.php?RecordID=416234, identifier: CRD42023416234.

## 1. Introduction

Carotid artery stenosis refers to the narrowing of the carotid artery lumen by ≥50% due to atherosclerosis, arterial dissection, arterial fibromuscular dysplasia, etc. Among them, carotid artery stenosis caused by atherosclerosis accounts for 90% of the total (1). According to whether there are relevant clinical symptoms, carotid artery stenosis can be divided into symptomatic carotid artery stenosis and asymptomatic carotid artery stenosis (2). A global analysis of the general population aged 30–79 shows that 1.5% of the total population have carotid artery stenosis with a stenosis rate of ≥50% (2). According to statistics, carotid artery stenosis is associated with 20% to 30% of ischemic stroke and is also an important factor for stroke recurrence (3). In addition, studies have found that carotid artery stenosis also increased the risk of myocardial infarction, coronary heart disease hospitalization, and cardiovascular death (4).

Carotid artery revascularization surgery is a way of treatment for severe asymptomatic carotid artery stenosis and moderate to severe symptomatic carotid artery stenosis, mainly including carotid endarterectomy (CEA) and carotid artery stenting (CAS) (5, 6). Revascularization surgery is a beneficial supplement to standard drug therapy, with the main purpose of improving the blood supply to the brain from the ipsilateral carotid artery. It is an important means of primary or secondary prevention of ischemic stroke (7). However, studies have shown that when the prevalence and mortality rate of perioperative complications are more than3%, the benefits of revascularization surgery will be offset (8). Therefore, how to reduce postoperative complications is an important issue in perioperative treatment and nursing.

Frailty, most commonly seen in the elderly, is a syndrome that reflects reduced physiological reserves and the accumulation of comorbidities, resulting from the gradual decline of multiple physiological systems and the body's inability to maintain homeostasis (9, 10). It can lead to a decrease in the patient's resistance to interference such as injury and disease, and cause a series of complications, leading to a significant increase in acute decompensation, functional decline, and death (11–14).

Pre-operative frailty refers to a state of frailty that is determined by the health care provider before surgery using validated frailty assessment tools (15–17). Patients with pre-operative frailty are different from normal frailty patients. On account of the influence of disease and the superposition of surgical stress and trauma, there is a higher incidence of pre-operative frailty and postoperative adverse outcome events (15). As the adverse effects of pre-operative frailty have been confirmed by numerous studies, an increasing of researchers have begun to focus on whether pre-operative frailty can affect the occurrence of postoperative complications of carotid artery revascularization, but an integrated quantitative analysis is currently lacking. Therefore, a meta-analysis of relevant evidence is necessary, which will provide a reference direction for reducing postoperative complications of carotid artery revascularization.

## 2. Materials and methods

### 2.1. Protocol and registration

This study was conducted based on the Preferred Reporting Items for Systematic and Meta-analysis (PRISMA) (18) and the Meta-analysis of Observational Studies in Epidemiology checklist (MOOSE) (19). The protocol has been registered with the International Prospective Systems Review Registry (Prospero) (registration number CRD42023416234).

### 2.2. Search strategy

We systematically searched for relevant research on Medline, Embase, Ovid, CINAHL, Web Of Science, and Cochrane Library from the establishment of the database to June 2023. The following MeSH terms or keywords were used: “frail elderly” OR “frail” OR “elderly assessment” OR “frail” OR “frail syndrome”; “carotid artery stenosis” OR “carotid endarterectomy” OR “carotid artery stenting” OR “carotid artery revascularization”. A detailed search strategy can be found in Supplementary-Material.

### 2.3. Eligibility criteria

The inclusion criteria are as follows: (1) Patients undergoing carotid artery revascularization surgery, including CEA and CAS (including transcarotid artery revascularization, TCAR); (2) A confirmed diagnosis of frailty was made by a validated frailty assessment tool before surgery; (3) Observational studies, including prospective or retrospective cohort studies; (4) The article reports the prevalence of pre-operative frailty in patients; (5) The article reports the impact of frailty on postoperative death/stroke/myocardial infarction/non-home discharge.

Exclusion criteria: (1) Incomplete data included in the study; (2) The language of this publication is not English; (3) An article report or review published in the form of comments, conference abstracts, and cases.

### 2.4. Data extraction

Two researchers independently extracted data from eligible articles, including research characteristics (first author, publication time, country, female, number of patients, number of frail patients, study design, frailty assessment tool, database, prevalence rate)and adverse postoperative outcomes (death, stroke, myocardial infarction, non-home discharge). During the data extraction process, two researchers should reach a consensus on the extracted data, if there are differences, they would discuss with the third researcher.

### 2.5. Risk of bias assessment and certainty of evidence

Two researchers used the Newcastle Ottawa Quality Scale (NOS) (20), an evaluation tool for cohort studies, to evaluate the risk of bias for the included study. According to the contents of the scale items, six areas were evaluated, such as participant selection, confounding variables, exposure measurement, outcome evaluator blind method, incomplete result date, and selective result reporting. The total score of the scale is 9 points, according to the score, the risk of bias included in the study is divided into three levels: 0–4 points for low-quality, 5–6 points for medium quality, and ≥7 points for high-quality studies. The quality of evidence for each conclusion was assessed using GRADE Working Group guidelines, which could be divided into four categories: low, medium and high.

### 2.6. Statistical analysis

Summarize the OR and 95% CI of postoperative outcome events related to pre-operative frailty and perform logarithmic transformation (logistic OR), then analyze the impact of pre-operative frailty on postoperative adverse outcomes using RevMan 5.4 software. Summarize the prevalence of frailty in patients using Stata 17.0 and conduct subgroupanalysis based on the study design. If the degree of frailty was stratified, the data would be uniformly combined. Use the chi-square heterogeneity test to evaluate overall heterogeneity results and express them as *I*^2^ statistics. If *I*^2 ^≥ 50% indicates heterogeneity, a random effects model would be used; Otherwise, use a fixed effects model.

## 3. Results

### 3.1. Study selection

**Figure 1:**
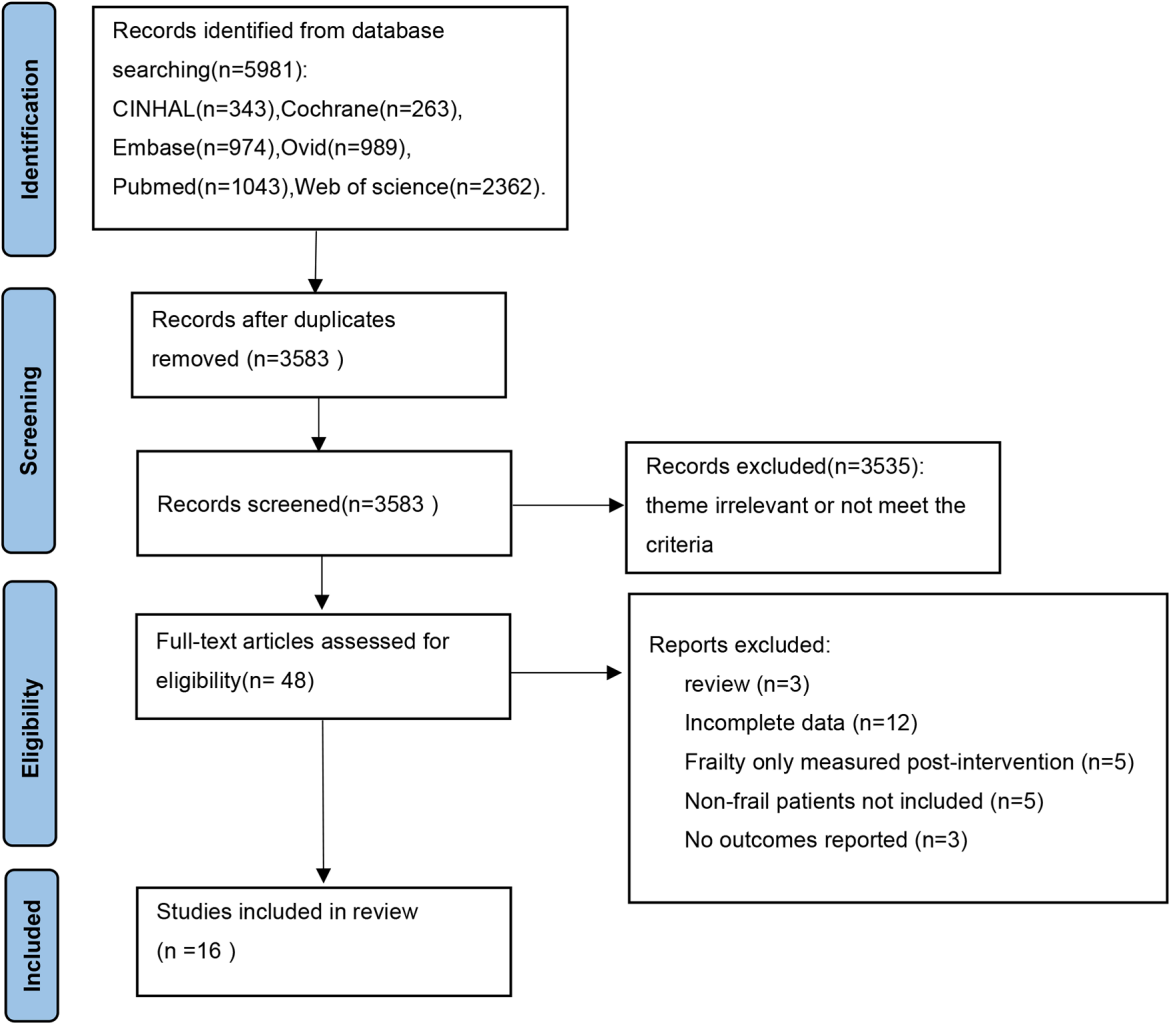
PRISMA flow chart of literature screening.

Through preliminary search, 5981 articles were obtained, and after screening based on inclusion and exclusion criteria, a total of 16 studies were included (PRISMA flow chart, Figure 1).

### 3.2. Characteristics of the included studies

A total of 16 studies involved a total of 1,692,338 patients. Among them, 5 (21–25) were prospective cohort studies, and 11 (26–36) were retrospective cohort studies. Of the 16 studies, 1 (21) came from Portugal, 1 (28) from Canada, 2 (22, 25) from the Netherlands, and 12 (23, 24, 26, 27, 29–36) from the United States. In these studies,10 studies (21–23, 25–27, 30, 33, 35, 36) included patients undergoing CEA surgery, 3 studies (24, 29, 31) included patients undergoing CAS surgery, and 3 studies (28, 32, 34) included patients undergoing CAS or CEA surgery. A total of seven frailty assessment tools were included in the study, with four studies using 5-item modified frailty index(mFI-5), four using Risk Analysis Index(RAI), two using 11-item modified frailty index(mFI-11), two using Clinical Frailty Scale(CFS), two using Groningen Frailty Indicator(GFI), one using Johns Hopkins Adjusted Clinical Groups frailty indicators(ACG), and one using Cardiovascular Health Study Index(CHS).The other features are detailed in Supplementary Table S1.

### 3.3. Risk of bias assessment and certainty of evidence

A total of 16 studies were included, with a literature quality score ranging from 6 to 9 points. The literature quality level is at the upper middle level, with 1 literature (24) scoring 9 points, 12 (21–23, 25–33) scoring 8 points, 2 (34, 36) scoring 7 points, and 1 (35) scoring 6 points. See Supplementary Table S2 for details. According to the results assessed by GRADE guidelines, the quality of the evidence for prevalence of pre-operative frailty in carotid artery revascularization was graded as “low”, increased mortality risk was graded as “high”, increased stroke risk was graded as “moderate”, increased myocardial infarction risk was graded as “moderate”, increased non-home discharge increased was graded as “high”. See Supplementary Table S3 for details.

### 3.4. Prevalence of pre-operative frailty

**Figure 2:**
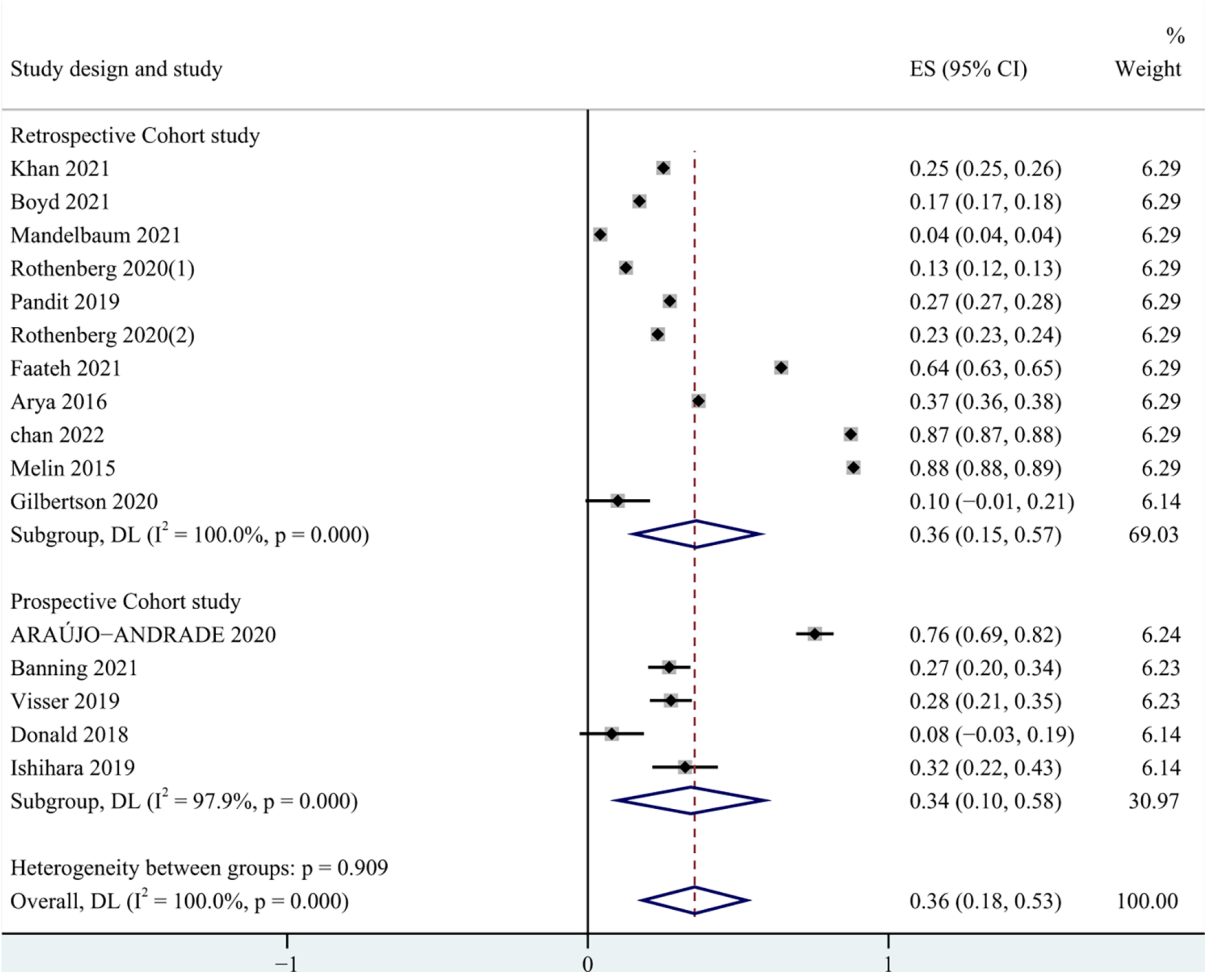
Prevalence of pre-operative frailty.

**Figure 3:**
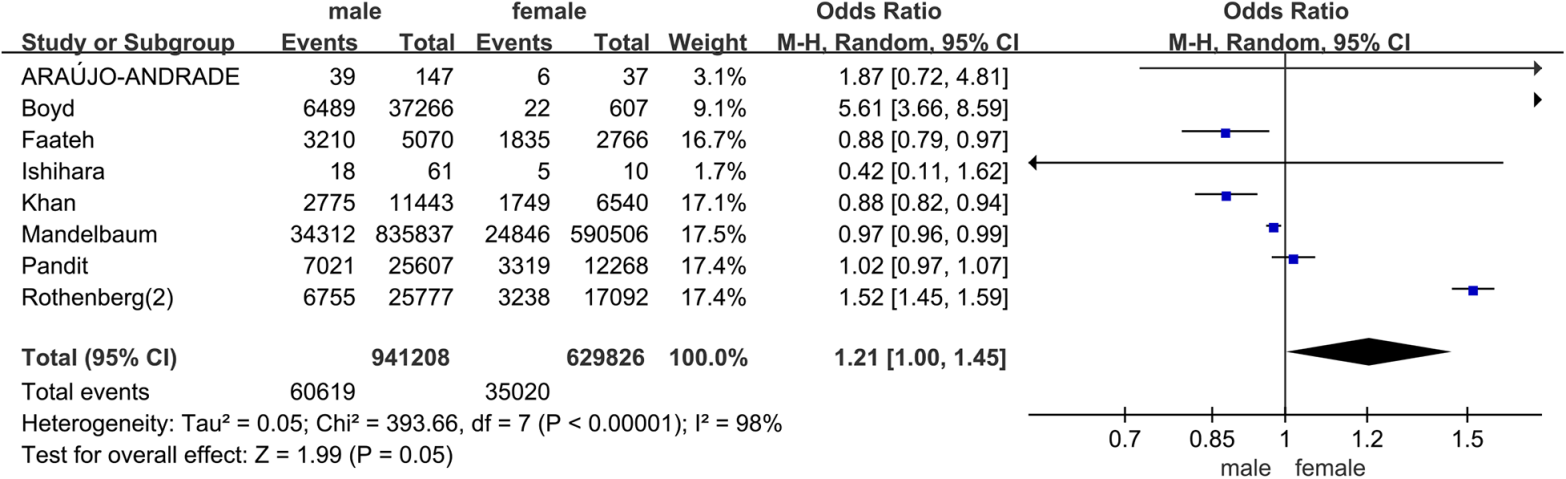
Sex difference in prevalence of pre-operative frailty.

In all studies, the prevalence of frailty ranged from 4.0% to 88.0%. Among patients undergoing carotid artery revascularization, the overall prevalence of frailty was 36.0% (95% CI = 0.18–0.53, *P* < 0.001). The results of the subgroup analysis showed that the prevalence of frailty was 34.0% (95% CI = 0.10–0.58, *P* < 0.001) in the prospective cohort study, and 36.0% (95% CI = 0.15–0.57, *P* < 0.001) in the retrospective cohort study. Due to significant heterogeneity between them (*I*^2^ = 100%, *P* < 0.001), a random effects model was used for analysis. See Figure 2 for details. In addition, in pooled analyses, we found that female were more likely than male to experience pre-operative frailty. See Figure 3 for details.

### 3.5. Mortality

**Figure 4:**
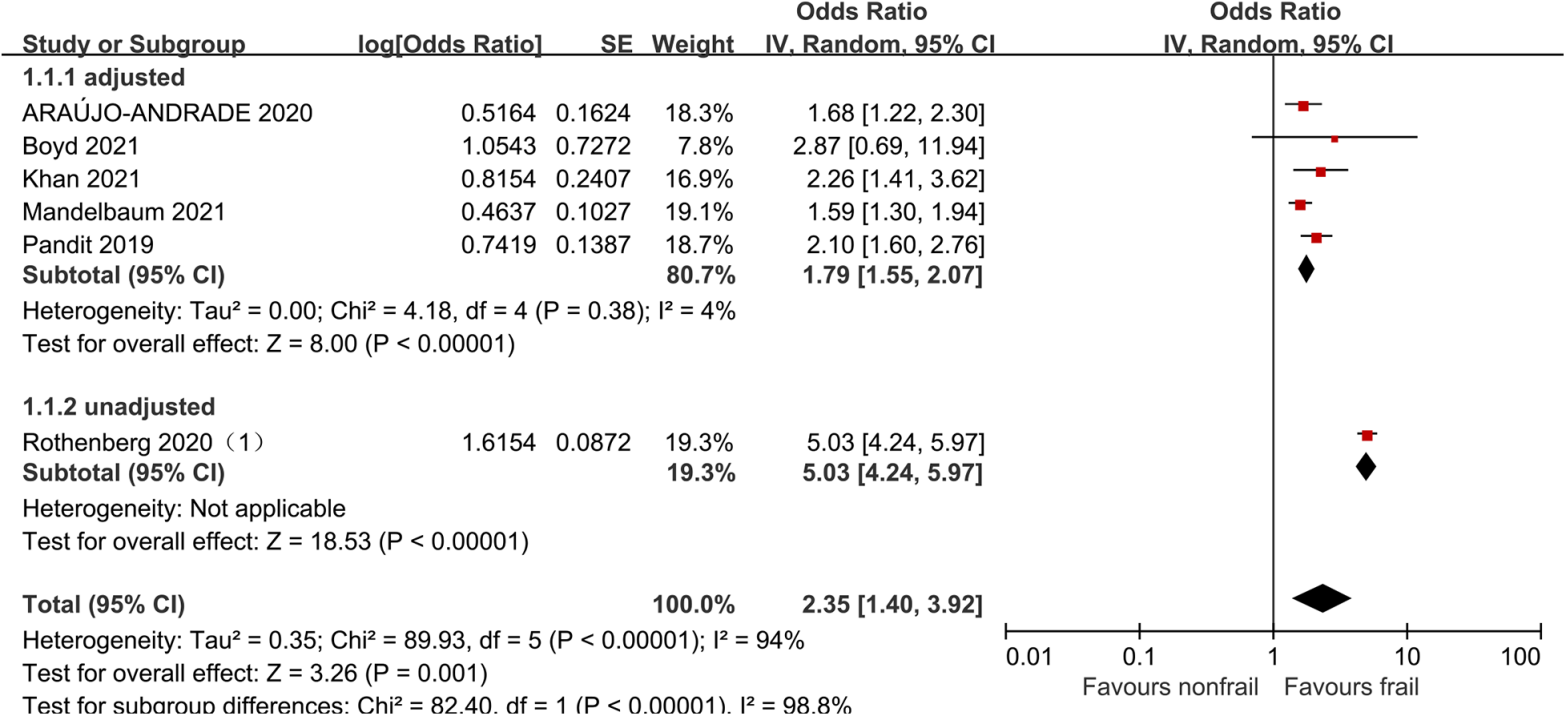
Effect of pre-operative frailty on mortality.

Six studies (21, 27, 31, 32, 34, 36) analyzed the impact of pre-operative frailty on postoperative mortality using a random effects model, and the results showed a statistically significant difference with an OR of 2.35 (95% CI = 1.40–3.92, *P* = 0.001, *I*^2^ = 94%). Due to significant heterogeneity, subgroup analysis was conducted based on whether the data was adjusted. Five studies adjusted were included (21, 27, 31, 32, 34), with an OR of 1.79 (95% CI = 1.55–2.07, *P* < 0.001, *I*^2^ = 4%). One study unadjusted was included (36), with an OR of 5.03 (95% CI = 4.24–5.97, *P* < 0.001). The results all showed statistically significant differences. See Figure 4 for details.

### 3.6. Stroke

**Figure 5:**
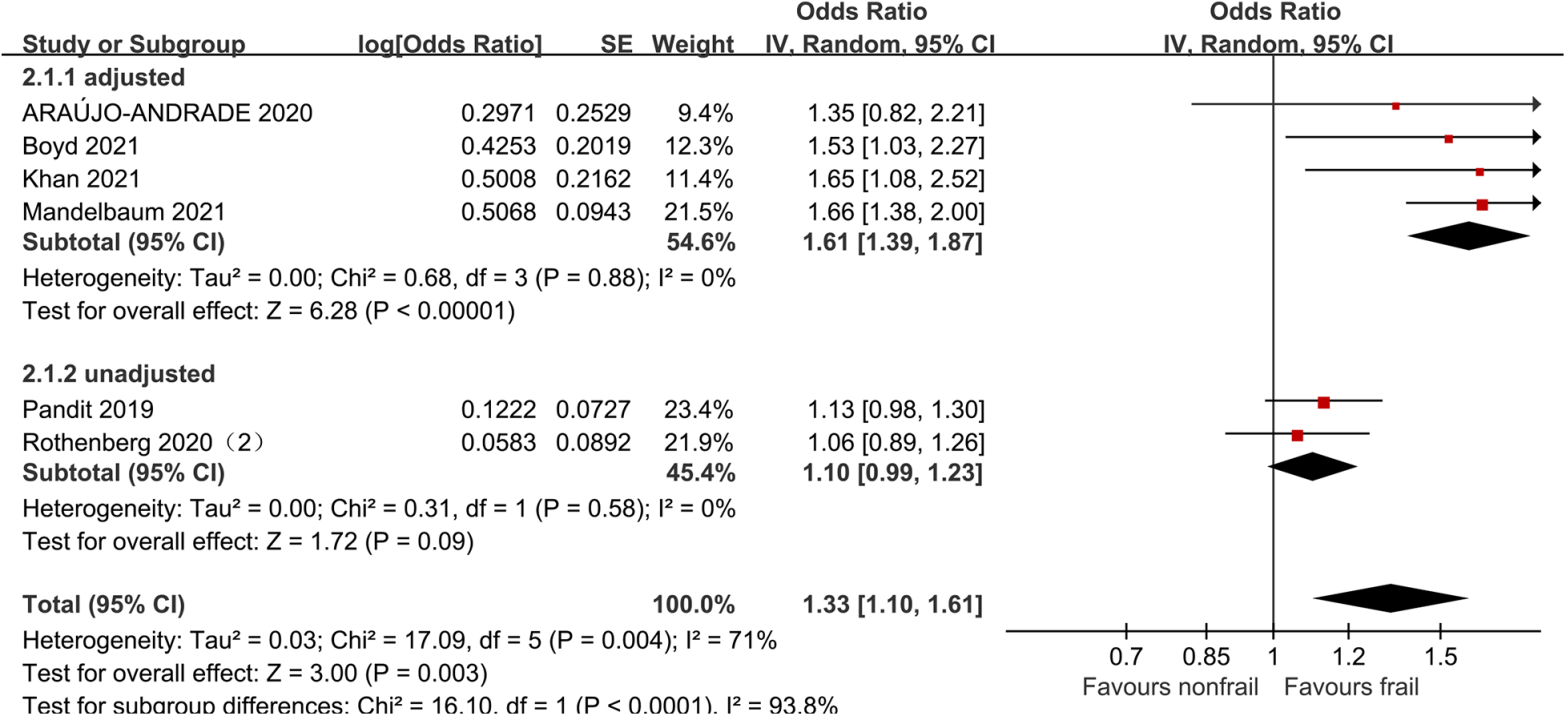
Effect of pre-operative frailty on stroke.

Six studies (21, 27, 31, 32, 34, 35) analyzed the impact of pre-operative frailty on postoperative stroke, using a random effects model. The results showed a statistically significant difference with an OR of 1.33 (95% CI = 1.10–1.61, *P* = 0.003, *I*^2^ = 71%). Due to significant heterogeneity, subgroup analysis was conducted based on whether the data was adjusted. Four studies adjusted were included (21, 27, 31, 32), and the results showed statistically significant differences (OR = 1.61, 95% CI = 1.39–1.87, *P* < 0.001, *I*^2^ = 0). Two studies unadjusted were included (34, 35), the results showed no statistically significant differences (OR = 1.10, 95% CI = 0.99–1.23, *P* = 0.09, *I*^2^ = 0). See Figure 5 for details.

### 3.7. Myocardial infarction

**Figure 6:**
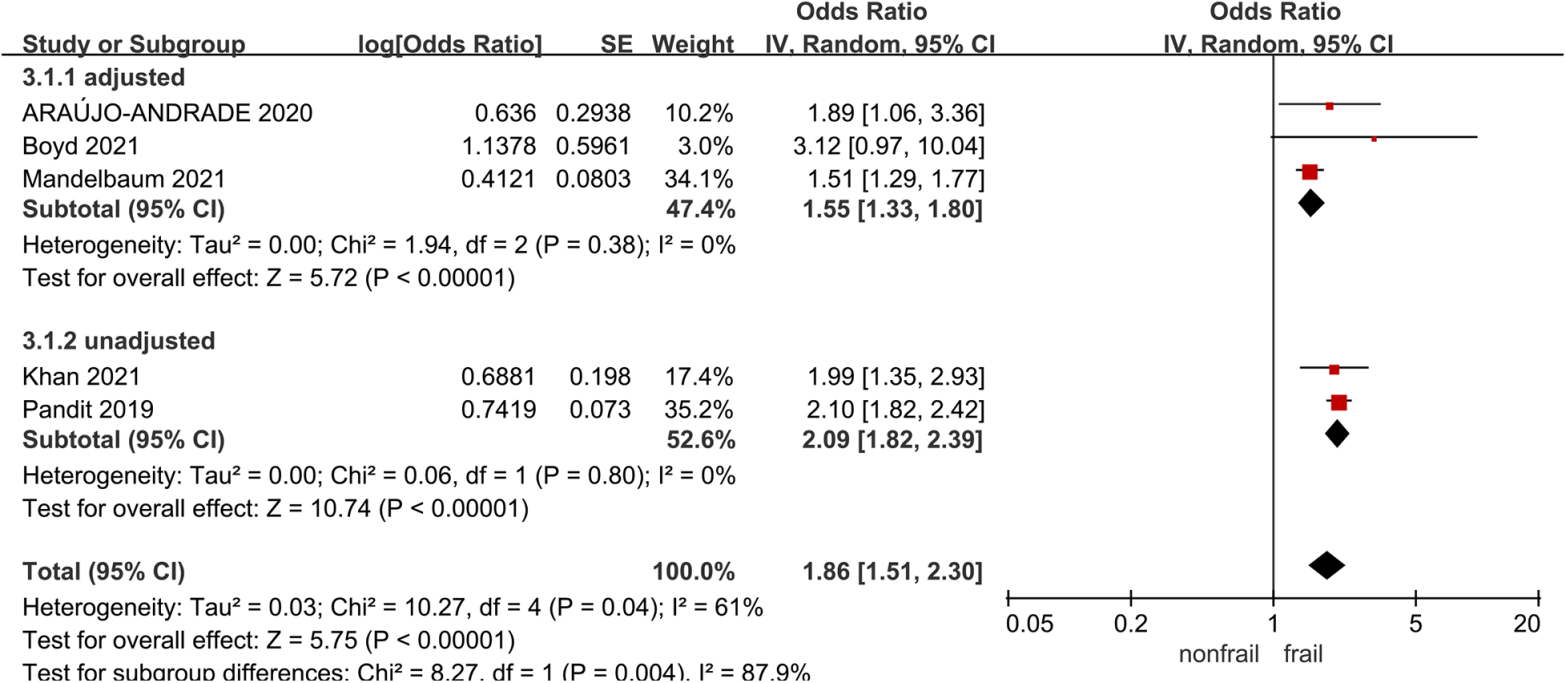
Effect of pre-operative frailty on myocardial infarction.

Five studies (21, 27, 31, 32, 34) analyzed the impact of pre-operative frailty on postoperative myocardial infarction, using a random effects model analysis. The results showed a statistically significant difference, with an OR of 1.86 (95% CI = 1.51–2.30, *P* < 0.001, *I*^2^ = 61%). Due to significant heterogeneity, subgroup analysis was conducted based on whether the data was adjusted. Three studies were included in the adjusted group (21, 27, 32) (OR = 1.55, 95% CI = 1.33–1.80, *P* < 0.001, *I*^2^ = 0), two studies were included in the unadjusted group (31, 34), with an OR of 2.09 (95% CI = 1.82–2.39, *P* < 0.001, *I*^2^ = 0).The results all showed statistically significant differences. See Figure 6 for details.

### 3.8. Non-home discharge

**Figure 7:**
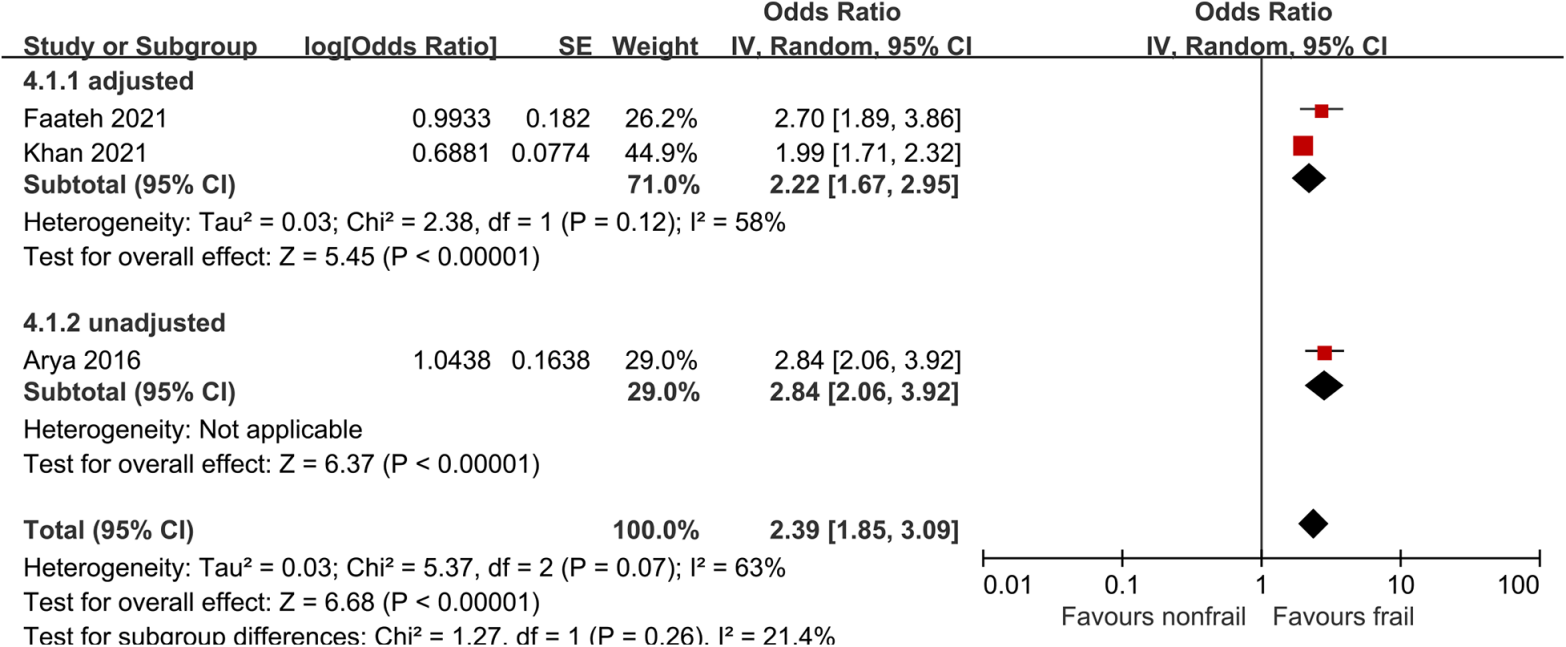
Effect of pre-operative frailty on non-home discharge.

Three studies (26, 29, 31) analyzed the impact of pre-operative frailty on postoperative non-home discharge, using a random effects model. The results showed a statistically significant (OR = 2.39, 95% CI = 1.85–3.09, *P* < 0.001, *I*^2^ = 63%). Due to significant heterogeneity, subgroup analysis was conducted based on whether the data was adjusted. Two studies were included in the adjusted group (29, 31), with an OR of 2.22 (95% CI = 1.67–2.95, *P* < 0.001, *I*^2^ = 58%).One unadjusted study was included in the adjusted group (26), with an OR of 2.84 (95% CI = 2.06–3.92, *P* < 0.001). The results all showed statistically significant differences. See Figure 7 for details.

### 3.9. Publication bias

A publication bias test was conducted on the prevalence of pre-operative frailty, and the results showed that there was a certain publication bias(Begg's test: *P* = 0.043 and Egger's test: *P* = 0.037). Due to the limited number of articles, publication bias testing was not conducted in other outcomes (37).

## 4. Discussion

This study showed that the prevalence of pre-operative frailty in carotid artery revascularization was 36.0%. Through subgroup analysis, it was found that the prevalence of frailty in prospective cohort studies was 34.0%, and in retrospective cohort studies, it was 36.0%. Patients with frailty have a higher risk of postoperative mortality, stroke, myocardial infarction, and non-home discharge compared to patients without frailty.

Recent researches showed that due to aging of body function, atherosclerosis, and other factors, patients undergoing carotid artery revascularization have a high degree of pre-operative frailty (38, 39). Banning (40) found that the prevalence of pre-operative frailty of CEA is 23.9%, lower than the results of this study, which may be related to the inclusion of CAS patients and expanded sample size in this study.

In this meta-analysis, we found that pre-operative frailty in carotid artery revascularization increases the risk of postoperative mortality (OR = 2.35, 95% CI = 1.40–3.92, *P* = 0.001, *I*^2^ = 94%). Karam (41) demonstrated that pre-operative frailty can predict postoperative mortality (OR = 2.06) among 67,308 hospitalized patients in vascular surgery, similar to the conclusions of this study.

The occurrence of postoperative stroke and myocardial infarction is an important indicator for evaluating the efficacy of carotid artery revascularization surgery. In this study, we found that the risk of postoperative stroke in frail patients was 1.33 times that of non-frail patients, and the risk of postoperative myocardial infarction was 1.83 times that of non-frail patients. Pre-operative frailty increased the risk of postoperative complications.

In addition, the important thing is that a good long-term prognosis is also a necessary condition for consideration of preventive treatment. Non-home discharge is closely related to an increased risk of complications, frequent readmissions, and death (42, 43). This study found that pre-operative frailty increased the risk of non-home discharge by 1.39-fold (OR = 2.39, 95% CI = 1.85–3.09, *P* < 0.001, *I*^2^ = 63%), which is similar to the study results of Ebrahimian (44).

The advantage of this meta-analysis is to focus on patients with carotid artery stenosis, as frailty and carotid artery stenosis are mutually causal and exacerbating. On the one hand, insufficient blood supply to the carotid artery and the combined effects of other cardiovascular diseases drive frailty (45). On the other hand, frailty also accelerates the change of carotid central structure and atherosclerosis, further exacerbating carotid stenosis (11). Patients with carotid artery stenosis who undergoing revascularization surgery require strong physical reserves as support during the surgery. For frail patients, the reduction of physiological reserves reduces their ability to resist intraoperative risks, leading to adverse postoperative outcomes and affecting their prognosis. Therefore, emphasizing perioperative frailty management is of great significance for the rehabilitation of patients with carotid artery stenosis.

The information obtained from pre-operative frailty assessment can be used to alert patients to surgical risks and develop personalized treatment plans, and frailty patients often tend to prefer less invasive or conservative treatment pathways (46). At the same time, clinical doctors and frail patients can jointly discuss the benefits of surgery, and make collaborative decisions centered on the patient, achieving the goal of balancing potential risks and improving quality of life.

Frailty can also be seen as a potential therapeutic goal, aimed at optimizing the patient's physical strength, nutrition, and functional status before and after surgery by managing frailty. The sustained stress experienced by frail patients during major surgeries can deplete their physiological reserves, leading to progressive decompensation after surgery. Early pre-habilitation can avoid the risk of partial loss in advance (47). For example, pre-operative physical exercise and enhanced nutrition or medication can be used to enhance body function to cope with the risks and trauma caused by surgery (48). Hall (49) found that through comprehensive frailty screening and early management of patients undergoing elective surgery, the mortality of frailty patients decreased from 12.2% to 3.8%, and treatment including ventilator management and postoperative dialysis was also better maintained.

Furthermore, frailty assessment can provide a reference for the allocation of nursing resources and the adjustment of nursing strategies for frail patients undergoing carotid artery revascularization. Frailty patients often combine physical, psychological, social, and other aspects of frailty into one (50). In postoperative care, in addition to connecting with pre-operative pre-rehabilitation, attention should also be paid to the comprehensive physical and mental recovery of patients. Research has shown that frail patients often do not want to face up to the "losses" caused by their frailty, believing that frailty will reduce their sense of self-identity, quality of life, or life goals, so often presenting different value needs (51). Therefore, frailty management requires the joint efforts of doctors, patients, and family members to explore the core values of patients. Only by understanding the optimal needs of frail patients can personalized nursing strategies and health education plans be better formulated.

There are many tools to assess frailty in clinical practice, medical staff should choose assessment tools that are in line with patients' conditions. For example, mFI scale has a wide range of assessment fields and high sensitivity, but it requires a large amount of clinical information, which is extremely tedious and time-consuming (52). However, the CFS scale shows a good psychometric property, a short assessment time, and does not require additional equipments. At present, there is evidence that the CFS scale tool is suitable for the assessment of cardiovascular disease patients, which can be further verified in the future (53). In addition, many studies define the evaluation results as binary cut-off, but the development of frailty is a dynamic process, and data analysis should reflect this continuity (54). So, presenting the results in the way of categorical and/or continuous fashion will have greater clinical significance.

The American Society of Surgeons National Surgical Quality Improvement Program (ACS NSQIP) and the American Geriatric Society (AGS) (55) recommend that all elderly surgical patients should have a frailty score record on admission. The National Confidential Enquiry into Patient Outcome and Death publication in the UK also pointed out that frailty needs to be considered as an important surrogate markers of risk in the perioperative period (56). However, in the fifth NELA report (57), it was pointed out that among frail patients aged 65 and above, only 36.9% of doctors provide medication to the elderly during the postoperative period, indicating that there is currently insufficient emphasis on frailty management in clinical practice and the need to further strengthen medical staff's awareness.

### 4.1. Limitations

There are also several shortcomings in this study. First of all, there is no strict limit on inclusion in the research database, and some data may overlap. Secondly, due to the diversity of assessment tools for frailty and the varying definitions of multiple frailties, it is not possible to uniformly define assessment tools. Finally, the prevalence of pre-operative frailty have heterogeneity and publication bias, so the interpretation of the results should be more cautious.

### 4.2. Future directions

This study has revealed the role of pre-operative frailty in adverse outcomes after carotid revascularization, and it can be further clarified whether it has different effects on surgical types in the future. It is also meaningful to explore the factors that influence pre-operative frailty in carotid artery revascularization, which can help us find more targeted ways to improve pre-operative frailty. In addition, some researchers have found that pre-operative frailty plays a different role in patients with symptomatic and asymptomatic carotid stenosis, but the evidence is limited now, so more studies are needed in the future.

## 5. Conclusion

In this study, we have found that the prevalence of pre-operative frailty in carotid artery revascularization was 36% and pre-operative frailty could increase the risk of mortality, stroke, myocardial infarction, and non-home discharge after carotid artery revascularization. In clinical practice, we should strengthen management strategies for pre-operative frailty to reduce the risk of adverse events after carotid artery revascularization.

## Data Availability

The original contributions presented in the study are included in the article/**Supplementary Material**, further inquiries can be directed to the corresponding author.
