# Supplementary material for: Prevalence and adverse outcomes of pre-operative frailty in patients undergoing carotid artery revascularization: a meta-analysis
Source: Front Cardiovasc Med. 2023 Nov 28;10:1297848. doi: 10.3389/fcvm.2023.1297848 (PMC10715064; doi:10.3389/fcvm.2023.1297848)
Supplement: Supplementary file 1 [file Table1.docx]

Supplementary Material

**Prevalence and adverse outcomes of pre-operative frailty in patients**

**undergoing carotid artery revascularization:a meta-analysis**

**Zeyu Liu, Ying Yao, Meiwan Zhang,Yan Ling,Xiaoyan Yao,Min Hu***

***Correspondence:** Min Hu: zm_mz2144@163.com

# Supplementary Tables

Systematic literature review search terms and strategy.

- 1. **Cochrane Library search strategies:**

| #1 | MeSH descriptor: [Carotid Stenosis] explode all trees | 920 |
| --- | --- | --- |
| #2 | (Carotid stenosis OR Carotid Stenos* OR Carotid thrombo* OR Carotid Artery Narrowing* OR Carotid Artery Plaque* OR Carotid Artery):ti,ab,kw (Word variations have been searched | 7564 |
| #3 | #1 or #2 | 7564 |
| #4 | MeSH descriptor: [Angioplasty] explode all trees | 5439 |
| #5 | MeSH descriptor: [Vascular Surgical Procedures] explode all trees | 19191 |
| #6 | (angioplasty OR Vascular Surgical Procedures OR carotid revascularization OR Carotid Artery Surgery OR carotid interventions OR endovascular procedure* OR endovascular surgery OR vascular surgery):ti,ab,kw (Word variations have been searched) | 25509 |
| #7 | #4 or #5 or #6 | 35585 |
| #8 | MeSH descriptor: [Endarterectomy, Carotid] explode all trees | 665 |
| #9 | MeSH descriptor: [Stents] explode all trees | 5563 |
| #10 | (carotid endarterectomy OR StentS OR CEA OR carotid artery stenting OR CAS OR transfemoral CAS OR transcarotid artery revascularization OR TCAR OR Transfemoral carotid artery stenting OR TFCAS):ti,ab,kw (Word variations have been searched) | 41691 |
| #11 | #8 or #9 or #10 | 41691 |
| #12 | MeSH descriptor: [Frail Elderly] explode all trees | 961 |
| #13 | MeSH descriptor: [Frailty] explode all trees | 458 |
| #14 | MeSH descriptor: [Geriatric Assessment] explode all trees | 1837 |
| #15 | (Frail elderly OR frailty OR geriatric assessment OR frail* OR Frailty Syndrome):ti,ab,kw (Word variations have been searched) | 10443 |
| #16 | #12 or #13 or #14 or #15 | 10443 |
| #17 | #3 and #7 | 3614 |
| #18 | #17 or #11 | 43617 |
| #19 | #18 and #16 | 153 |
|  |  |  |

- 1. **Embase search strategies:**

| #7 | #5 AND #6 | 225 |
| --- | --- | --- |
| #6 | 'frail elderly':ab,ti OR frailty:ab,ti OR | 55,462 |
|  | 'geriatric assessment':ab,ti OR frail*:ab,ti OR |  |
| #5 | #3 OR #4 | 160,425 |
| #4 | 'carotid endarterectomy':ab,ti OR stents:ab,ti OR cea:ab,ti OR 'carotid artery stenting':ab,ti OR cas:ab,ti OR 'transfemoral cas':ab,ti OR 'transcarotid artery revascularization':ab,ti OR tcar:ab,ti OR 'transfemoral carotid artery stenting':ab,ti OR tfcas:ab,ti | 156,623 |
| #3 | #1 AND #2 | 7,845 |
| #2 | angioplasty:ab,ti OR 'vascular surgical procedures':ab,ti OR 'carotid revascularization':ab,ti OR 'carotid artery surgery':ab,ti OR 'carotid interventions':ab,ti OR 'endovascular procedure*':ab,ti OR 'endovascular surgery':ab,ti OR 'vascular surgery':ab,ti | 96,233 |
| #1 | 'carotid stenosis':ab,ti OR 'carotid stenos*':ab,ti OR 'carotid thrombo*':ab,ti OR 'carotid artery narrowing*':ab,ti OR 'carotid artery plaque*':ab,ti OR 'carotid artery':ab,ti | 96,443 |
|  |  |  |

- 1. **Medline search strategies:**

| 1 | exp Carotid Stenosis/ | | | 17838 |
| --- | --- | --- | --- | --- |
| 2 | (Carotid stenosis or Carotid Stenos* or Carotid thrombo* or Carotid Artery Narrowing* or Carotid Artery Plaque* or Carotid Artery).mp. | | | 94533 |
| 3 | exp Angioplasty/ | | | 64145 |
| 4 | exp Vascular Surgical Procedures/ | | | 288986 |
| 5 | (angioplasty or Vascular Surgical Procedures or carotid revascularization or Carotid Artery Surgery or carotid interventions or endovascular procedure* or endovascular surgery or vascular surgery).mp. | | | 147572 |
| 6 | exp Endarterectomy, Carotid/ | | | 9668 |
| 7 | exp Stents/ | | | 88756 |
| 8 | (carotid endarterectomy or StentS or CEA or carotid artery stenting or CAS or transfemoral CAS or transcarotid artery revascularization or TCAR or Transfemoral carotid artery stenting or TFCAS).mp. | | | 227956 |
| 9 | exp Frail Elderly/ | | | 14750 |
| 10 | exp Frailty/ | | | 8384 |
| 11 | exp Geriatric Assessment/ | | | 32026 |
| 12 | (Frail elderly or frailty or geriatric assessment or frail* or Frailty Syndrome).mp. | | | 65561 |
| 13 | 1 or 2 | | | 94533 |
| 14 | 3 or 4 or 5 | | | 311453 |
| 15 | 6 or 7 or 8 | | | 229805 |
| 16 | 9 or 10 or 11 or 12 | | | 65561 |
| 17 | 13 and 14 | | | 21769 |
| 18 | 15 or 17 | | | 238000 |
| 19 | 16 and 18 | | | 236 |
|  | |  |  | |

- 1. **CINHAL search strategies:**

| S7 | S4 AND S6 | 76 |
| --- | --- | --- |
| S6 | S3 OR S5 | 182,767 |
| S5 | S1 AND S2 | 14,554 |
| S4 | SU Frail elderly OR frailty OR geriatric assessment OR frail* OR Frailty Syndrome | 105,796 |
| S3 | SU carotid endarterectomy OR StentS OR CEA OR carotid artery stenting OR CAS OR transfemoral CAS OR transcarotid artery revascularization OR TCAR OR Transfemoral carotid artery stenting OR TFCAS | 175,364 |
| S2 | SU angioplasty OR Vascular Surgical Procedures OR carotid revascularization OR Carotid Artery Surgery OR carotid interventions OR endovascular procedure* OR endovascular surgery OR vascular surgery | 171,014 |
| S1 | SU Carotid stenosis OR Carotid Stenos* OR Carotid thrombo* OR Carotid Artery Narrowing* OR Carotid Artery Plaque* OR Carotid Artery | 180,680 |
|  |  |  |

- 1. **Pubmed search strategies:**

| 7 | ((((carotid stenosis[MeSH Terms]) OR (Carotid stenosis[Title/Abstract] OR Carotid Stenos*[Title/Abstract] OR Carotid thrombo*[Title/Abstract] OR Carotid Artery Narrowing*[Title/Abstract] OR Carotid Artery Plaque*[Title/Abstract] OR Carotid Artery[Title/Abstract])) AND (((angioplasty[MeSH Terms]) OR (Vascular Surgical Procedures[MeSH Terms])) OR (angioplasty[Title/Abstract] OR Vascular Surgical Procedures[Title/Abstract] OR carotid revascularization[Title/Abstract] OR Carotid Artery Surgery[Title/Abstract] OR carotid interventions[Title/Abstract] OR endovascular procedure*[Title/Abstract] OR endovascular surgery[Title/Abstract] OR vascular surgery[Title/Abstract]))) OR (((carotid endarterectomy[MeSH Terms]) OR (StentS[MeSH Terms])) OR (carotid endarterectomy[Title/Abstract] OR StentS[Title/Abstract] OR CEA[Title/Abstract] OR carotid artery stenting[Title/Abstract] OR CAS[Title/Abstract] OR transfemoral CAS[Title/Abstract] OR transcarotid artery revascularization[Title/Abstract] OR TCAR[Title/Abstract] OR Transfemoral carotid artery stenting[Title/Abstract] OR TFCAS[Title/Abstract]))) AND ((((Frail elderly[MeSH Terms]) OR (frailty[MeSH Terms])) OR (geriatric assessment[MeSH Terms])) OR (Frail elderly[Title/Abstract] OR frailty[Title/Abstract] OR geriatric assessment[Title/Abstract] OR frail*[Title/Abstract] OR Frailty Syndrome[Title/Abstract])) | 203 |
| --- | --- | --- |
| 6 | (((carotid stenosis[MeSH Terms]) OR (Carotid stenosis[Title/Abstract] OR Carotid Stenos*[Title/Abstract] OR Carotid thrombo*[Title/Abstract] OR Carotid Artery Narrowing*[Title/Abstract] OR Carotid Artery Plaque*[Title/Abstract] OR Carotid Artery[Title/Abstract])) AND (((angioplasty[MeSH Terms]) OR (Vascular Surgical Procedures[MeSH Terms])) OR (angioplasty[Title/Abstract] OR Vascular Surgical Procedures[Title/Abstract] OR carotid revascularization[Title/Abstract] OR Carotid Artery Surgery[Title/Abstract] OR carotid interventions[Title/Abstract] OR endovascular procedure*[Title/Abstract] OR endovascular surgery[Title/Abstract] OR vascular surgery[Title/Abstract]))) OR (((carotid endarterectomy[MeSH Terms]) OR (StentS[MeSH Terms])) OR (carotid endarterectomy[Title/Abstract] OR StentS[Title/Abstract] OR CEA[Title/Abstract] OR carotid artery stenting[Title/Abstract] OR CAS[Title/Abstract] OR transfemoral CAS[Title/Abstract] OR transcarotid artery revascularization[Title/Abstract] OR TCAR[Title/Abstract] OR Transfemoral carotid artery stenting[Title/Abstract] OR TFCAS[Title/Abstract])) | 166100 |
| 5 | ((carotid stenosis[MeSH Terms]) OR (Carotid stenosis[Title/Abstract] OR Carotid Stenos*[Title/Abstract] OR Carotid thrombo*[Title/Abstract] OR Carotid Artery Narrowing*[Title/Abstract] OR Carotid Artery Plaque*[Title/Abstract] OR Carotid Artery[Title/Abstract])) AND (((angioplasty[MeSH Terms]) OR (Vascular Surgical Procedures[MeSH Terms])) OR (angioplasty[Title/Abstract] OR Vascular Surgical Procedures[Title/Abstract] OR carotid revascularization[Title/Abstract] OR Carotid Artery Surgery[Title/Abstract] OR carotid interventions[Title/Abstract] OR endovascular procedure*[Title/Abstract] OR endovascular surgery[Title/Abstract] OR vascular surgery[Title/Abstract])) | 18633 |
| 4 | (((Frail elderly[MeSH Terms]) OR (frailty[MeSH Terms])) OR (geriatric assessment[MeSH Terms])) OR (Frail elderly[Title/Abstract] OR frailty[Title/Abstract] OR geriatric assessment[Title/Abstract] OR frail*[Title/Abstract] OR Frailty Syndrome[Title/Abstract]) | 65480 |
| 3 | ((carotid endarterectomy[MeSH Terms]) OR (StentS[MeSH Terms])) OR (carotid endarterectomy[Title/Abstract] OR StentS[Title/Abstract] OR CEA[Title/Abstract] OR carotid artery stenting[Title/Abstract] OR CAS[Title/Abstract] OR transfemoral CAS[Title/Abstract] OR transcarotid artery revascularization[Title/Abstract] OR TCAR[Title/Abstract] OR Transfemoral carotid artery stenting[Title/Abstract] OR TFCAS[Title/Abstract]) | 159582 |
| 2 | ((angioplasty[MeSH Terms]) OR (Vascular Surgical Procedures[MeSH Terms])) OR (angioplasty[Title/Abstract] OR Vascular Surgical Procedures[Title/Abstract] OR carotid revascularization[Title/Abstract] OR Carotid Artery Surgery[Title/Abstract] OR carotid interventions[Title/Abstract] OR endovascular procedure*[Title/Abstract] OR endovascular surgery[Title/Abstract] OR vascular surgery[Title/Abstract]) | 311359 |
| 1 | (carotid stenosis[MeSH Terms]) OR (Carotid stenosis[Title/Abstract] OR Carotid Stenos*[Title/Abstract] OR Carotid thrombo*[Title/Abstract] OR Carotid Artery Narrowing*[Title/Abstract] OR Carotid Artery Plaque*[Title/Abstract] OR Carotid Artery[Title/Abstract]) | 75493 |
|  |  |  |

- 1. **Web of science search strategies:**

| #1 | TS=(Carotid stenosis OR Carotid Stenos* OR Carotid thrombo* OR Carotid Artery Narrowing* OR Carotid Artery Plaque* OR Carotid Arter | 183681 |
| --- | --- | --- |
| #2 | TS=(angioplasty OR Vascular Surgical Procedures OR carotid revascularization OR Carotid Artery Surgery OR carotid interventions OR endovascular procedure* OR endovascular surgery OR vascular surgery | 567231 |
| #3 | TS=(carotid endarterectomy OR StentS OR CEA OR carotid artery stenting OR CAS OR transfemoral CAS OR transcarotid artery revascularization OR TCAR OR Transfemoral carotid artery stenting OR TFCAS ) | 314106 |
| #4 | TS=(Frail elderly OR frailty OR geriatric assessment OR frail* OR Frailty Syndrome) | 95606 |
| #5 | #1 AND #2 | 61242 |
| #6 | #3 OR #5 | 352667 |
| #7 | #6 AND #4 | 375 |
|  |  |  |
